# Infrared Aircraft Detection Algorithm Based on High-Resolution Feature-Enhanced Semantic Segmentation Network

**DOI:** 10.3390/s24247933

**Published:** 2024-12-11

**Authors:** Gang Liu, Jiangtao Xi, Chao Ma, Huixiang Chen

**Affiliations:** 1 College of Information Engineering, Henan University of Science and Technology, Luoyang 471023, China; 9906066@haust.edu.cn (C.M.); 17518898227@163.com (H.C.); 2 School of Electrical, Computer and Telecommunications Engineering, University of Wollongong, Wollongong, NSW 2522, Australia; jiangtao@uow.edu.au

**Keywords:** infrared aircraft, interference, target detection, location attention feature fusion network, hybrid atrous spatial pyramid pooling, dice loss, high-resolution semantic segmentation

## Abstract

In order to achieve infrared aircraft detection under interference conditions, this paper proposes an infrared aircraft detection algorithm based on high-resolution feature-enhanced semantic segmentation network. Firstly, the designed location attention mechanism is utilized to enhance the current-level feature map by obtaining correlation weights between pixels at different positions. Then, it is fused with the high-level feature map rich in semantic features to construct a location attention feature fusion network, thereby enhancing the representation capability of target features. Secondly, based on the idea of using dilated convolutions to expand the receptive field of feature maps, a hybrid atrous spatial pyramid pooling module is designed. By utilizing a serial structure of dilated convolutions with small dilation rates, this module addresses the issue of feature information loss when expanding the receptive field through dilated spatial pyramid pooling. It captures the contextual information of the target, further enhancing the target features. Finally, a dice loss function is introduced to calculate the overlap between the predicted results and the ground truth labels, facilitating deep excavation of foreground information for comprehensive learning of samples. This paper constructs an infrared aircraft detection algorithm based on a high-resolution feature-enhanced semantic segmentation network which combines the location attention feature fusion network, the hybrid atrous spatial pyramid pooling module, the dice loss function, and a network that maintains the resolution of feature maps. Experiments conducted on a self-built infrared dataset show that the proposed algorithm achieves a mean intersection over union (mIoU) of 92.74%, a mean pixel accuracy (mPA) of 96.34%, and a mean recall (MR) of 96.19%, all of which outperform classic segmentation algorithms such as DeepLabv3+, Segformer, HRNetv2, and DDRNet. This demonstrates that the proposed algorithm can achieve effective detection of infrared aircraft in the presence of interference.

## 1. Introduction

In infrared imaging-based aircraft target detection, on the one hand, the target is situated within a complex natural background environment such as the ground, clouds, and sea clutter. The infrared radiation intensity significantly attenuates after long-distance transmission, resulting in a small target size on the imaging plane, with blurred shape features and a low signal-to-noise ratio in the image. On the other hand, the target may release interference similar to its own radiation, shape, and motion characteristics, and sometimes the interference may partially or even completely obscure the target. In situations where interference partially obscures the target, although conditions for recognition at the overall level are not present, certain parts of the aircraft, such as the nose, cockpit, propellers, engine, and tail, may still be exposed. This makes partial recognition possible, which is of great significance for inferring the type and location of the parts of the aircraft obscured by the interference. Infrared aircraft target detection in complex natural backgrounds and artificial interference is a highly challenging research topic.

According to different feature extraction approaches, the methods for recognizing infrared aircraft in complex natural backgrounds and artificial interference can be divided into traditional methods and deep learning-based methods. Traditional methods that precisely model target features recognize targets based on characteristics such as color, texture, grayscale, and contour under certain conditions [1]. However, when environmental changes exceed the predefined rules, these traditional methods become ineffective. Compared to traditional methods, deep learning-based methods do not require predefined feature extraction rules and have the capability to autonomously learn features. They are widely used in tasks such as image classification, object detection, and semantic segmentation. Semantic segmentation involves detecting objects at the pixel level, assigning a semantic category label to each pixel in the image. Target detection tasks based on semantic segmentation use convolutional neural networks to assign a category label to each pixel in the image, distinguishing between the target and the background. Target detection is achieved by utilizing the position and category of each pixel. This approach mainly includes techniques such as fully convolutional dilated semantic segmentation, fully convolutional symmetric (encoder–decoder) semantic segmentation, feature fusion, and attention mechanisms [2,3].

Reference [4] proposes a fully convolutional neural network (FCN) that replaces the fully connected layers in the VGG-16 network with convolutional layers, and integrates both shallow and deep features to enhance feature representation. However, the pooling layers in the FCN can cause the loss of positional information for some pixels, and the segmentation process does not fully consider the contextual information of the pixels. One technical approach to solving this problem is dilated semantic segmentation based on a fully convolutional network, with the DeepLab series being a classic algorithm. This method can increase the receptive field without increasing the number of parameters. Reference [5] integrates convolutional neural networks with probabilistic graphical models to form the Deeplabv1 network, and introduces atrous convolution to enlarge the receptive field of feature maps. Reference [6] introduces the atrous spatial pyramid pooling (ASPP) module into the DeepLabv1 network to form Deeplabv2. This module captures multi-scale feature information and performs feature fusion, which is advantageous for detecting objects at different scales. Reference [7] proposes DeepLabv3, which modifies the ASPP structure by concatenating and parallelizing atrous convolutions to better capture multi-scale feature information. Reference [8] integrates the encoder–decoder module and the Xception network into the DeepLabv3 architecture, proposing the DeepLabv3+ semantic segmentation network. This enhancement allows for better understanding of contextual semantic information in images. Researchers both domestically and internationally have further developed research on object detection tasks starting from the DeepLab series algorithms. Reference [9] proposes an improved ASPP structure based on DeepLabv3+, incorporating heterogeneous receptive field fusion, and applies it to the segmentation and recognition of infrared smoke areas. Reference [10] proposes a two-stage model that combines the DeepLabv3+ semantic segmentation network with an improved YOLOv5 object detection network. This model is used to detect cow eyes and udders in thermal infrared images against complex backgrounds, and is applied to classify the severity of mastitis in cows. Reference [11] proposes a real-time infrared pedestrian segmentation method using DeepLabv3+ as its foundation. This method features a feature extraction network composed of spatial and contextual pathways. Reference [12] integrates the convolutional block attention module (CBAM) into DeepLabv3+ to improve the segmentation accuracy of armored targets in complex infrared battlefield scenarios. Reference [13] proposes an improved DeepLabv3+ algorithm based on dense connected ASPP to achieve precise segmentation of infrared images of electrical equipment defects. Reference [14] applies ResNet101 as the backbone network in DeepLabv3+ to achieve thermal fault recognition of power system components. Reference [15] integrates a ResNet-50 decoder into the DeepLabv3+ structure to effectively recognize malignant structures in breast ultrasound images, promoting diagnostic capabilities. In summary, the ASPP module in semantic segmentation utilizes dilated convolutions with different dilation rates to capture and fuse multi-scale feature information, which benefits the detection of targets at various scales.

Semantic segmentation networks with symmetric structure are another important method to address the issue of loss of pixel spatial position and semantic information caused by pooling. These networks, also known as encoder–decoder-based networks, work by convolution and pooling steps in the encoder to extract image features, and deconvolution and upsampling steps in the decoder to restore a series of pixel features of the image. Reference [16] proposes the U-Net algorithm for medical image semantic segmentation using a symmetric encoder–decoder structure. The encoder captures spatial and semantic information through convolution and downsampling, while the decoder restores feature map resolution through upsampling. Reference [17] employs an encoder–decoder structure to supplement infrared target detail information into the top-down high-level semantic feedback path for object detection. However, the dual-branch algorithm requires more memory during the training phase. Reference [18] proposes a method for stitching infrared images of wind turbine blades based on drone flight data and U-Net. This method utilizes the U-Net semantic segmentation network to remove complex backgrounds while preserving the complete regions of the blades to be stitched. Reference [19] proposes a defect detection method for composite materials using infrared thermography based on a semantic segmentation network. This method constructs a 3D U-Net network by employing a 3D convolution module and a temporal convolution module to extract the temporal and spatial features of thermal information. Reference [20] applies the U-Net algorithm to accurately segment infrared thermal images containing different types of buildings. This provides support for urban planners in identifying hotspots and cold spots in the city at different times, thereby aiding in strategy formulation. Reference [21] proposes a dual-channel semantic segmentation algorithm based on U-Net for multimodal remote sensing image segmentation. This algorithm separately extracts color space features from RGB remote sensing images and shape space features from near-infrared images, then concatenates the two types of features to achieve correlation and complementarity between the features of different modalities. Reference [22] constructs the RespathU-Net semantic segmentation network based on the U-Net framework by redesigning the encoder network structure, expanding the receptive field, and connecting residual paths. This network is applied to online defect detection in infrared thermography during the laser cladding process. Reference [23] proposes a dual U-shaped feature extraction network for fine segmentation of small infrared targets. The larger U-shaped network captures semantic information, while the smaller U-shaped network retains more positional information. Reference [24] proposes a dual-stream multi-scale network (DMU-Net) for urban building extraction based on U-Net. This network effectively fuses image features from different spectral bands. However, the drawback of the symmetric structured semantic segmentation network is that it has a large number of training parameters, high computational demands, and cannot achieve real-time segmentation.

The technology route of semantic segmentation based on feature fusion involves enhancing target features by fusing features from different layers of the feature extraction network in various ways. Based on the idea of feature fusion in Reference [25], the pyramid pooling module (PPM) and parallel multi-scale global pooling are used to aggregate contextual information from different regions, enhancing the target feature information. Reference [26] proposes image cascade network (ICNet), which introduces the cascade feature fusion unit to achieve real-time segmentation while ensuring accuracy. Reference [27] proposes the strip pooling network (SPNet) segmentation algorithm, which integrates strip and hybrid pooling to achieve the segmentation of objects with different shapes. In the strip pooling module, the input image is first processed with both horizontal and vertical strip pooling, resulting in two feature maps for each input image. These two feature maps are then fused together. Reference [28] uses a multi-scale fusion strategy to enhance target feature representation while reducing background interference, thereby improving the performance of infrared small target segmentation. Reference [11] integrates the semantic information of spatial and contextual paths through a feature fusion module. In order to address the issue of unstructured road detection at night based on thermal infrared images, Reference [29] proposes a dual-branch real-time segmentation network (URTSegNet), which includes a detail branch and a semantic branch. A simple and efficient aggregation block, designed using a gating mechanism, is employed to fuse the feature information from the two branches. Reference [30] proposes the HRNet network for human pose estimation, where convolutional layers of different resolution are connected in parallel, allowing for information exchange and fusion among them. During the final prediction, only the output of the high-resolution sub-network is used. Building on this, Reference [31] further proposes the HRNetv2 network for semantic segmentation, which aggregates feature layers of different resolution at the end of the network. Overall, the technology route based on feature fusion can effectively enhance target features, leading to improved segmentation results.

Incorporating attention mechanism into semantic segmentation network can capture crucial parts of semantic information, thereby enhancing the training efficiency of the segmentation network. Reference [32] introduces the dual attention network (DANet), which utilizes ResNet as the backbone network. It incorporates its output into two parallel self-attention networks to capture spatial and channel dependencies separately. The outputs of these self-attention networks are fused together before segmentation is performed. Reference [33] proposes the height-driven attention network (HANet) after incorporating self-attention mechanism, specifically designed for semantic segmentation in urban scenes with significant contextual variations. Reference [34] focuses on small target detection of ocean eddies by embedding a residual attention module into the U-Net network. This allows the model to pay more attention to the contour information of ocean eddies. Reference [35] integrates a global spatial attention module into the semantic segmentation network, which is designed for detecting buried objects in infrared images. This allows the model to focus on target regions and reduce interference from the background in the infrared images. Reference [36] addresses the semantic segmentation problem of clouds and shadows by proposing a hybrid branch semantic segmentation network composed of a convolutional network and transformer in parallel. It effectively captures long-range dependency through a self-attention mechanism. Reference [37] uses ResNet as the backbone and constructs a new cross-operation fusion attention module within a dual encoder–decoder model. This module effectively integrates features from two modal inputs, RGB and thermal infrared images, enabling semantic segmentation in autonomous driving environments. Reference [38] proposes a semantic segmentation algorithm that combines visible light and infrared images for the same problem. The algorithm designs two parallel encoders, and the decoder segments the fused image from the encoders. It employs a residual attention module within each branch to explore and enhance spatial features across multiple channels. Reference [39] introduces a graph neural network model for semantic segmentation of infrared images, based on top-down guided attention and gradient alignment. The model utilizes a guided attention module to address ambiguity in semantic encoding.

Due to the weak characteristics of infrared aircraft and the further reduction of learnable features when interference obscures the target, convolutional neural networks cannot sufficiently learn the features. Therefore, measures need to be taken to enhance the target features in semantic segmentation-based object detection models. Semantic segmentation based on feature fusion enhances target features by merging different levels of features or global and local features. Therefore, this paper uses the HRNetv2 network, which maintains the resolution of the input feature maps and fuses features from different resolution layers, as the baseline. Additionally, we incorporate the attention mechanism and dilated convolution as feature enhancement strategies to propose a new infrared aircraft detection algorithm based on semantic segmentation. Furthermore, in the process of infrared target detection, the imbalance between positive and negative samples can lead to false detections by the model. It is necessary to enhance the adequate learning of the small number of positive samples that contain target information. The dice loss function [40] calculates the overlap between the predicted result and the ground truth label to deeply extract foreground information, thereby enhancing the learning of the detection algorithm on small samples. Therefore, this paper proposes a high-resolution feature-enhanced semantic segmentation network (HFSSNet) to achieve the detection of infrared aircraft. The main contributions are as follows:

(1) A location attention feature fusion network (LAFFN) is designed. Through the location attention mechanism, the network captures the correlation weights between different location pixels to enhance the current level feature maps. These enhanced feature maps are then fused with high-level feature maps rich in semantic information, resulting in enhanced feature maps that contain both detailed and semantic information.
(2) A hybrid atrous spatial pyramid pooling (HASPP) module is designed to address the issue of losing feature information when expanding the receptive field with atrous spatial pyramid pooling. This is achieved by utilizing a serial structure of small atrous rate convolutions. HASPP can effectively capture the target’s contextual information and further enhance target features.
(3) To address the imbalance between infrared aircraft pixels and background pixels, the dice loss function is introduced to deeply explore foreground information. This guides the model to train towards increasing the overlap area between predicted result and ground truth label, thereby achieving thorough learning of the available samples.
(4) Combining the location attention feature fusion network, hybrid atrous spatial pyramid pooling module, dice loss function, and the network maintaining feature map resolution, this paper constructs an infrared aircraft detection algorithm based on high-resolution feature-enhanced semantic segmentation network.

The remaining parts of this paper are structured as follows: Section 2 will elaborate on the fundamental principles of the infrared aircraft detection algorithm based on high-resolution feature-enhanced semantic segmentation network. This includes the location attention feature fusion network, hybrid atrous spatial pyramid pooling, dice loss function, and the method for integrating with HRNetV2. Section 3 will present the experimental design and results analysis conducted on a custom-made infrared aircraft dataset. Finally, conclusions and prospects for future research will be provided.

## 2. The Design of High-Resolution Feature-Enhanced Semantic Segmentation Network

The high-resolution feature-enhanced semantic segmentation network (HFSSNet), based on the idea of preserving the resolution of input feature maps and enhancing target feature representation through the fusion of feature layers at different resolution, selects HRNetv2 as the foundational framework for the semantic segmentation algorithm. HRNetv2 mainly consists of four parts: the high-resolution stage, the low-resolution stage, the horizontal stage, and the merge stage. The high-resolution stage contains multiple residual modules [41] to extract target feature information, outputting feature maps with the same resolution as the input. The low-resolution stage performs the strided convolution on the feature maps output by the high-resolution stage to reduce the model’s memory usage. The horizontal stage introduces multiple parallel branches on feature maps of different resolution, facilitating interaction between these feature maps. The merge stage restores feature maps of different resolution to the same size as the high-resolution feature map through convolution and upsampling. Subsequently, all outputting feature maps are fused and fed into the softmax function for final classification.

**Figure 1:**
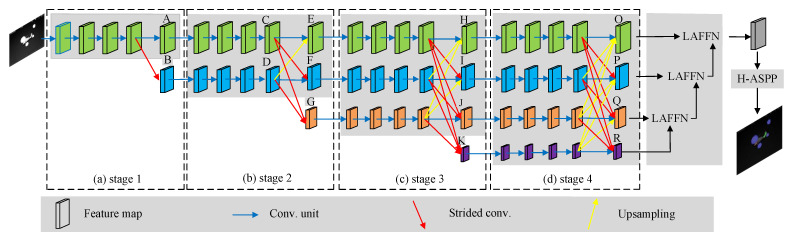
HFSSNet architecture diagram.

HFSSNet further enhances infrared aircraft features based on the HRNetv2 framework. Firstly, the feature maps outputted by HRNetv2 are inputted into a location attention feature fusion network. This network enhances the current level feature maps using location attention mechanism and integrates them with high-level feature maps rich in semantic information. The result is enhanced feature maps that contain detailed spatial and semantic information. Then, following the principle of maintaining receptive field size in feature extraction with dilated convolution, a concatenated structure of dilated convolution with a small dilation rate is utilized. This approach addresses the issue of information loss in infrared aircraft features caused by atrous spatial pyramid pooling. In addition, to address the issue of imbalanced pixel distribution between infrared aircraft and background pixels, dice loss is introduced to facilitate comprehensive learning of the target. The overall network structure of HFSSNet is illustrated in Figure 1.

### 2.1. HRNetv2 Network Structure

To address the issue of small target size in infrared aircraft detection, this paper selects the high-resolution network HRNetv2 as the main framework. HRNetv2 is a deep learning architecture proposed by Microsoft Research Asia. The core idea is to maintain image resolution to prevent the loss of target feature information and to fuse feature maps of different resolutions to obtain feature information at various resolutions, thereby improving the model’s ability to extract target features. The specific structure is as follows:

(1) Figure 1a includes a feature extraction branch, which inputs image $X\in R^{3\times H\times W}$ into four repeated residual modules. It then performs downsampling with strides of 1 and 4 to obtain feature maps $A\in R^{18\times H\times W}$ and $B\in R^{36\times (H/4)\times (W/4)}$. Here, *H* and *W* represent the height and width of the feature map, respectively.

(2) Figure 1b includes two feature extraction branches, where feature map *A* serves as the input for the first branch, and feature map *B* serves as the input for the second branch. Both are then input into four repeated residual modules, followed by downsampling with a stride of 1 to obtain feature maps $C\in R^{18\times H\times W}$ and $D\in R^{36\times (H/4)\times (W/4)}$. Next, the information fusion layer adjusts the resolution and the number of channels of feature maps *C* and *D* to be the same. Then, feature maps *C* and *D* undergo downsampling with strides of 8 and 2, respectively. Finally, they are cross-fused to obtain feature maps $E\in R^{18\times H\times W}$, $F\in R^{36\times (H/4)\times (W/4)}$, and $G\in R^{72\times (H/8)\times (W/8)}$.

(3) Figure 1c includes three feature extraction branches, with inputs corresponding to ***E***, ***F***, and ***G***. This section is similar to (2), and it ultimately outputs feature maps $H\in R^{18\times H\times W}$, $I\in R^{36\times (H/4)\times (W/4)}$, $J\in R^{72\times (H/8)\times (W/8)}$, and $K\in R^{144\times (H/16)\times (W/16)}$.

(4) Figure 1d includes four feature extraction branches, with inputs corresponding to ***H***, ***I***, ***J***, and ***K***. This section is similar to (3), resulting in feature maps $O\in R^{18\times H\times W}$, $P\in R^{36\times (H/4)\times (W/4)}$, $Q\in R^{72\times (H/8)\times (W/8)}$, and $R\in R^{144\times (H/16)\times (W/16)}$. Unlike (3), after obtaining feature maps ***O***, ***P***, ***Q***, and ***R***, the resolution and number of channels of feature maps ***P***, ***Q***, and ***R*** are adjusted to match those of feature map ***O***. Then, these four feature maps are concatenated to generate the final fused feature map, which is passed through a softmax layer for classification.

### 2.2. Location Attention Feature Fusion Network

**Figure 2:**
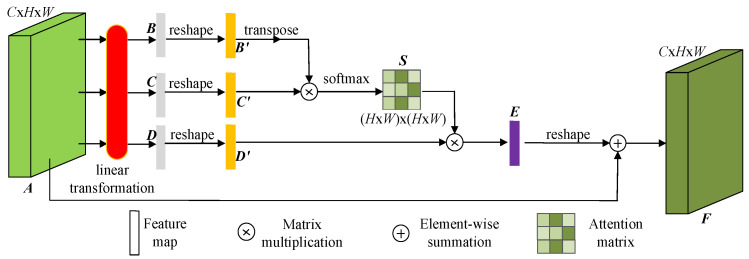
The structure of LAM.

The key to accurately segmenting infrared aircraft from images using deep convolutional neural network is the ability to fully learn target features. However, the infrared aircraft has weak features, and when interference obstructs the target, the learnable features are further reduced. This often increases the difficulty for convolutional neural networks to learn these features, thus necessitating feature enhancement for infrared aircraft. A commonly used method for feature enhancement is to introduce an attention mechanism in the deep learning convolutional neural network. The attention mechanism [42] originates from the varying degrees of human attention to different positions of objects, and can currently be divided into channel attention, spatial attention, and others. Channel attention primarily focuses on the relationship between different channels, without considering the local structure within the channels, which may overlook details of the local spatial information. Spatial attention can capture the spatial structure of local regions in the input data, allowing the algorithm to better understand the relationship between local pixels. Additionally, spatial attention focuses on the spatial positions in the input data rather than specific channels, which helps enhance the algorithm’s ability to handle geometric transformations [43]. However, it may overlook global contextual information. This paper designs a location attention mechanism (LAM) based on spatial attention to capture the correlation between pixels at different positions in the input feature map, balancing both local and global information, thereby enhancing the feature representation of the target. LAM first performs convolutional mapping on the input feature map to obtain different feature maps and then transforms their dimensions. Next, it calculates the correlation scores through matrix dot product between the feature maps to generate weight coefficients. Finally, it multiplies the weight coefficients with the reshaped feature maps and adds them to the input feature map to obtain the feature map with enhanced feature representation. The structure of LAM is shown in Figure 2.

The specific working principle of LAM is as follows:

(1) To obtain the feature information at different positions of the input feature map A, a linear transformation is applied to A, mapping it respectively to feature maps B,C,D, $B,C,D\in R^{C\times H\times W}$. To facilitate computation, the three-dimensional feature map B,C,D is reshaped into a two-dimensional feature map B′,C′,D′,$B\prime ,C\prime ,D\prime \in R^{C\times N}$,N=H×W.

(2) The correlation score is obtained by performing matrix multiplication between the transpose of B′ and C′, followed by using the softmax function to compute the positional attention weights, as shown in Equation (1).

(1) $$S_{ij}=\frac{exp(B\prime_{i}^{T}\cdot C\prime_{j})}{\sum_{j=1}^{N}exp(B\prime_{i}^{T}\cdot C\prime_{j})}$$

In the equation, $B\prime^{T}$ represents the transpose of matrix B′, $B\prime_{i}^{T}$ is the element at the *i*-th position of matrix $B\prime^{T}$, $C\prime_{j}$ represents the element at the *j*-th position of matrix C′, and $S_{ij}$ is the weight of the *i*-th position element relative to the *j*-th position element.

(3) The feature map $E\in R^{C\times N}$, which enhances pixel correlation, is generated by multiplying matrix D′ with weight $S_{ij}$, as shown in Equation (2).

(2) $$E_{j}=\sum_{i=1}^{N}\left(D\prime_{i}\cdot S_{ij}\right)$$

In the equation, $D\prime_{i}$ is the element at the *i*-th position of matrix D′, $E_{j}$ represents the element at the *j*-th position of matrix E.

(4) The enhanced feature map $F\in R^{C\times H\times W}$ is obtained by reshaping the feature map E and adding it to the original feature map A to further supplement the feature information, as shown in Equation (3).

(3) F=E+A

**Figure 3:**
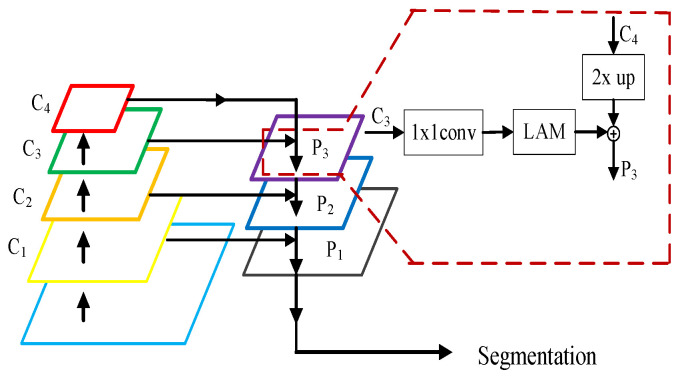
Location attention feature fusion network.

In the fusion stage of the HRNetv2 network framework, shown in Figure 1d, different resolution feature maps are fused by upsampling and adjusting the number of channels using 1 × 1 convolutions, without considering the dependencies between elements of the feature maps. Therefore, a location attention mechanism is introduced into the fusion stage in Figure 1d to construct the location attention feature fusion network (LAFFN), as shown in Figure 3.

In the structure of the location attention feature fusion network, the fusion process of the feature maps satisfies Equation (4):

(4) $$P_{i}=f_{LAM}(Conv_{1x1}(C_{i}))+Upsample(C_{i+1})$$

In the equation, Upsample represents upsampling, $f_{LAM}$ is the location attention mechanism, $Conv_{1x1}$ represents the 1 × 1 convolution, and $C_{i}$ is the feature maps, where *i* = 1, 2, 3. The specific construction process is as follows:

(1) The feature map $C_{3}$, downsampled 8 times by the backbone network, is first adjusted in channel number using a 1 × 1 convolution. It is then enhanced through LAM. After that, the feature map $C_{4}$, downsampled 16 times by the backbone network, is upsampled and fused with the enhanced feature map, resulting in the feature map $P_{3}$.

(2) The feature map $C_{2}$, downsampled 4 times by the backbone network, is first adjusted in channel number using a 1 × 1 convolution. It is then enhanced through LAM. After that, the feature map $P_{3}$ is upsampled and fused with the enhanced feature map, resulting in the feature map $P_{2}$.

(3) The feature map $C_{1}$, which has the same resolution as the input image, is first adjusted in channel number using a 1 × 1 convolution. It is then enhanced through LAM. After that, the feature map $P_{2}$ is upsampled and fused with the enhanced feature map, resulting in the feature map $P_{1}$. The aggregated feature map $P_{1}$ contains features from different scales, which helps the algorithm to better detect the target.

### 2.3. Hybrid Atrous Spatial Pyramid Pooling

LAFFN primarily aggregates infrared aircraft features from feature maps of different resolution. Furthermore, obtaining contextual information with varying receptive fields from a single feature map helps the algorithm detect infrared aircraft of different scales. Using larger convolutional kernels to expand the receptive field is one way to obtain contextual information from feature maps, but larger kernels increase computational complexity and reduce the resolution of the feature maps. Dilation convolution can expand the receptive field without increasing computational complexity or reducing the resolution of the feature maps. Therefore, this paper adopts the atrous spatial pyramid pooling (ASPP) structure and designs the hybrid atrous spatial pyramid pooling (HASPP) module to extract contextual information from the last layer of feature maps $P_{1}$ in the LAFFN, thereby enhancing the target feature representation furtherly.

**Figure 4:**
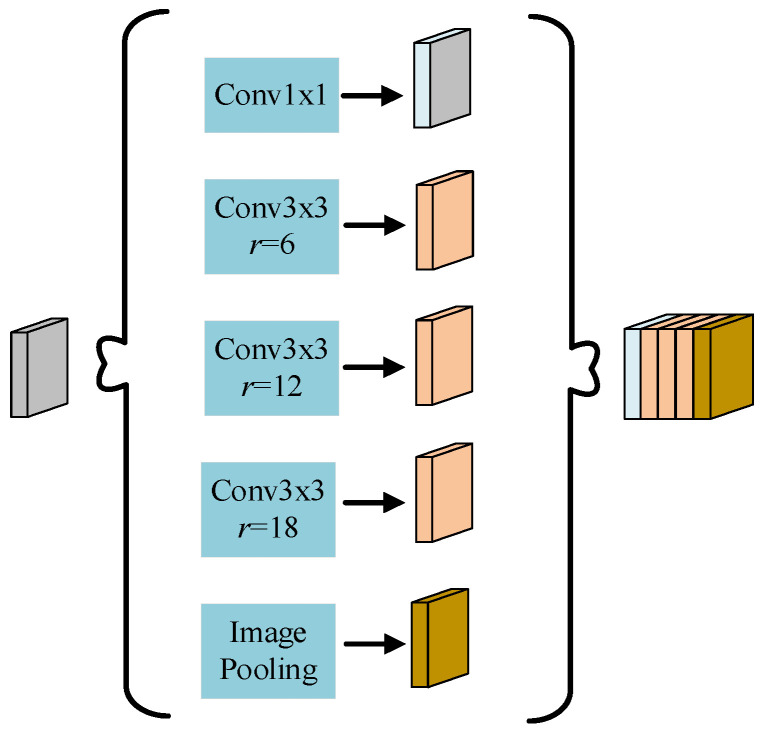
Atrous spatial pyramid pooling.

The ASPP structure, as shown in Figure 4, employs atrous convolutions with different dilation rates to capture contextual information from different receptive fields of the feature map [6]. However, atrous convolutions with dilation rates of 6, 12, and 18 tend to easily lose feature information when detecting small objects.

**Figure 5:**
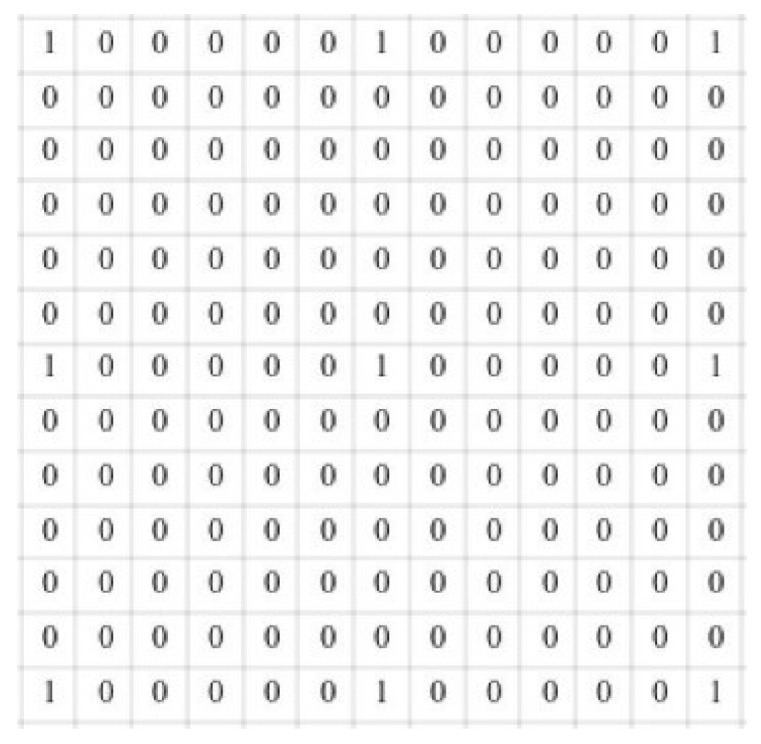
Dilated convolution kernel.

The structure of a 2D atrous convolution with kernel size k=3 and dilation rate r=6 is shown in Figure 5. From the figure, it can be observed that only nine pixels participate in the computation each time, resulting in a loss of feature information.

For this issue, Reference [8] densely connects different feature maps in the ASPP module, while Reference [44] uses dilated convolutions with [1, 3, 6, 9] small dilation rates in a parallel structure within the ASPP module. Although both improve the ASPP module to enhance feature utilization, they do not completely solve the problem of feature information loss. Reference [45] proposes a design criterion for the dilation rate of dilated convolution:

(5) $$M_{2}\leq k_{2}$$

(6) $$M_{i}=\max[M_{i+1}–2r_{i},2r_{i}–M_{i+1},r_{i}]$$

In the formula, $k_{2}$ represents the kernel size of the second layer in the serial structure, *i* denotes the index of the dilated convolution in the serial structure, $r_{i}$ signifies the dilation rate of the dilated convolution set in the serial structure, and $M_{i}$ indicates the maximum distance between non-zero values. In the final layer of the serial structure, $M_{n}=r_{n}$.

**Figure 6:**
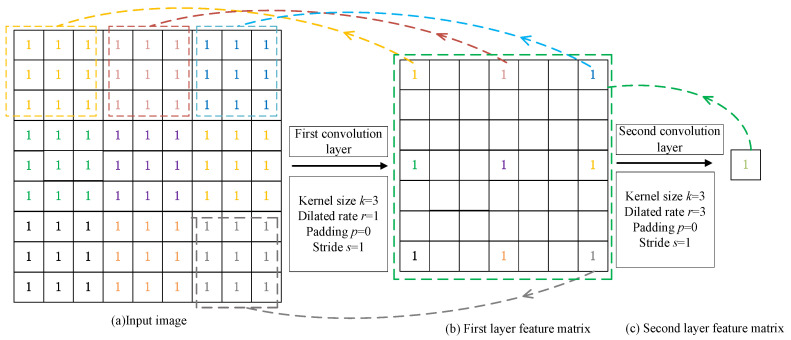
Calculation diagram of HASPP serial structure.

Reference [45] points out that when the dilation rate of the dilated convolution meets the above criteria, feature information will not be lost. The detailed calculation process of feature extraction is illustrated using a serial structure with dilation rates *r* = 1 and *r* = 3 for dilated convolutions, as shown in Figure 6.

Assume that Figure 6a is a 9 × 9 two-dimensional grayscale image. Figure 6b is the first layer feature map obtained by applying the ordinary convolution with kernel k=3 on Figure 6a. Figure 6c is the second layer feature map obtained by applying the dilated convolution with kernel k=3 and dilation rate r=3 on Figure 6b. The formulas for calculating the size of the dilated convolution kernel and the receptive field after dilated convolution are shown in Equations (7) and (8):

(7) k′=r×(k−1)+1

(8) $$l_{n}=\left\{\begin{array}{cc} l_{n-1}+(k_{n}-1){\prod_{i=1}^{n-1}s}_{i} & n\geq 2\\ k_{1} & n=1 \end{array}\right\}$$

In these equations, r is the dilation rate of the dilated convolution, k denotes the size of the convolution kernel before dilation, k′ is the size of the convolution kernel after dilation, $l_{n-1}$ represents the size of the receptive field at layer n−1, $k_{n}$ is the size of the convolution kernel at layer n, and $s_{i}$ is the stride of the convolution kernel.

From Equations (7) and (8), when the convolution kernel is k=3 and the dilation rate is r=3, the receptive field for each element in Figure 6c is 7 × 7 in Figure 6b. Only the nine elements in Figure 6b marked with the number one participate in the calculation. Each element in Figure 6b has a receptive field of 3 × 3 in Figure 6a, and any element in Figure 6b is computed from the nine elements of the same color in Figure 6a. By analyzing Figure 6a, it can be found that when the ordinary convolution with kernel k=3 is used in the first layer and the dilated convolution with kernel k=3 and dilation rate r=3 is used in the second layer, the input image’s feature information is not lost.

Therefore, this paper designs the HASPP module based on the criteria defined in Equations (5) and (6). ASPP uses a parallel structure of dilated convolution with dilation rates [1, 6, 12, 18]. Based on the principle that the receptive field is not reduced in feature extraction using dilated convolution, the dilated convolution with kernel k=3 and dilation rate r=18 in ASPP can be transformed into a serial structure of dilated convolutions with dilation rates [1, 3, 5, 9], [1, 7, 11], and so on. Substituting the dilation rates [1, 3, 5, 9] into Equation (6) yields:

(9) $$M_{3}=\max[M_{4}–2r_{3},2r_{3}–M_{4},r_{3}]=\max[9–10,10–9,5]=5$$

(10) $$M_{2}=\max[M_{3}–2r_{2},2r_{2}–M_{3},r_{2}]=\max[5–6,6–5,3]=3$$

At this point, $M_{2}=3\leq k=3$ satisfies Equation (5), and the target feature information will not be lost.

Substituting the dilation rates [1,7,11] into Equation (6) yields:

(11) $$M_{2}=\max[M_{3}–2r_{2},2r_{2}–M_{3},r_{2}]=\max[11–14,14–11,7]=7$$

At this point, $M_{2}=7\geq k=3$ does not satisfy Equation (5), and the target feature information will be lost.

**Figure 7:**
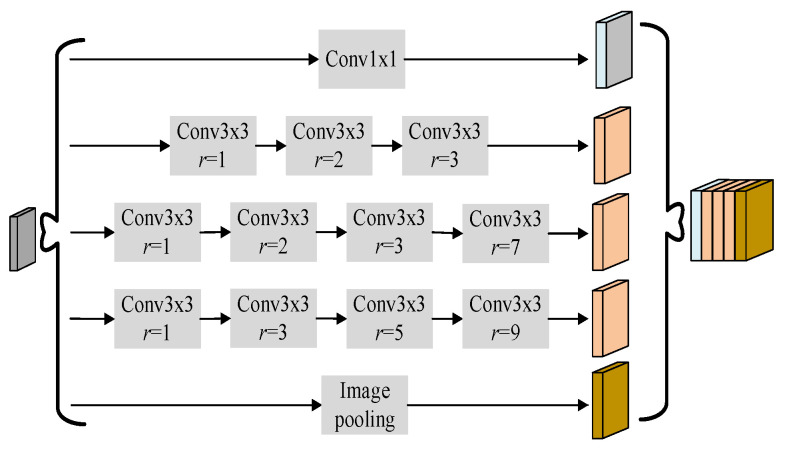
Hybrid atrous spatial pyramid pooling.

Therefore, the dilated convolution with kernel k=3 and dilation rate r=18 in ASPP should be converted to a serial structure of dilated convolutions with dilation rates [1, 3, 5, 9]. Similarly, the dilated convolutions in ASPP with kernel k=3 and dilation rate r=12, as well as kernel k=3 and dilation rate r=6, can be converted into the serial structures of dilated convolutions with dilation rates [1, 2, 3] and [1, 2, 3, 7], respectively. The designed HASPP structure is shown in Figure 7.

### 2.4. Loss Function Design

To alleviate the issue of false detection in segmentation algorithms caused by the imbalance between infrared aircraft pixels and background pixels, this paper introduces the dice loss function [40] to calculate the overlap between the predicted result and the ground truth label, thereby deeply mining the foreground information. Dice coefficient is a metric function used to evaluate the similarity between two samples. A higher value indicates greater similarity between the samples, as shown in Equation (12):

(12) $$Dice=\frac{2|X\cap Y|}{\left|X|+|Y\right|}$$

In the equation, X represents the pixel label of the ground truth image, Y is the pixel label of the predicted image, |X∩Y| is the number of pixels in the intersection of the predicted and ground truth images, and |X| and |Y| denote the number of pixels in X and Y respectively. The dice loss expression is shown in Equation (13):

(13) $$Dice\;Loss=1-\frac{2|X\cap Y|}{\left|X|+|Y\right|}$$

A larger dice loss value indicates a smaller overlap area between the predicted result and the ground truth label. To reduce this loss value, the dice loss function deeply mines the foreground information, guiding the model to train in a direction that increases the overlap area between the predicted result and the ground truth label, thereby achieving thorough learning from the existing samples.

The total loss of the high-resolution semantic segmentation network constructed in this paper consists of the cross-entropy loss and the dice loss. The cross-entropy loss is used for pixel classification and prediction, while the dice loss is used to deeply mine the foreground information. The expression is shown in Equation (14):

(14) $$Loss=-\frac{1}{N}\;(\sum_{i=1}^{N}\sum_{j=1}^{M}(y_{i}\log(p_{i}))+1-\frac{2|X\cap Y|}{\left|X|+|Y\right|}$$

In the equation, i=[1,⋯,N] is the index for the number of image pixels, j=[1,⋯,M] is the index for the number of classes, $y_{i}$ indicates whether the pixel label is correctly recognized (with $y_{i}$ = 1 for correct recognition and $y_{i}$ = 0 otherwise), and $p_{i}$ represents the probability of the pixel being correctly predicted.

## 3. Experiments and Analysis

### 3.1. Experimental Environment and Parameter Configuration

The operating system for the experiments in this paper is Windows 10. The CPU model is Intel(R) Core(TM) i7-10700 CPU @ 2.90 GHz, with a RAM of 32 GB. The GPU model is NVIDIA 3090, with a VRAM of 24 GB. CPU is from Intel Corporation in Santa Clara, CA, USA. GPU is from NVIDIA Corporation in Santa Clara, CA, USA.

All algorithms are built and run based on the pytorch 1.8.0 framework, using python 3.7 as the programming language. CUDA 11.3.1 and CUDNN 8.2.1 which are developed by NVIDIA Corporation are used to accelerate the GPU. During the training phase, the batch size is set to 8 and the SGD optimizer is used, with a learning rate of 0.004 and a weight decay of 0.0001. The training is conducted for a total of 100 epochs, with the learning rate gradually decreasing linearly each epoch.

### 3.2. Dataset Construction

**Figure 8:**
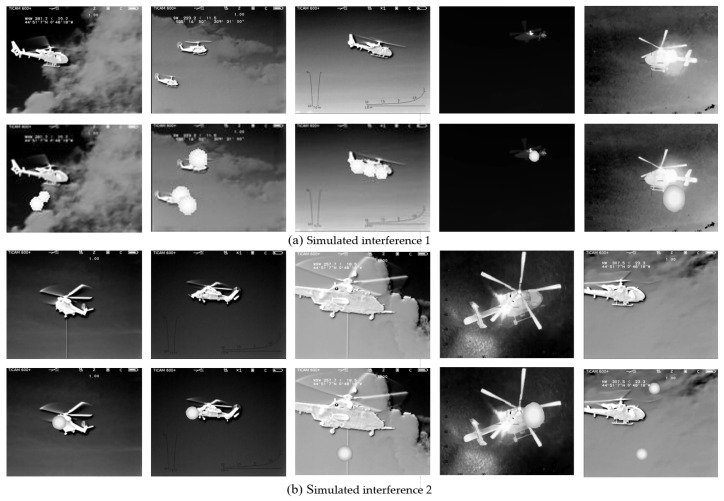
Infrared aircraft image with simulated interference under sky background.

**Figure 9:**
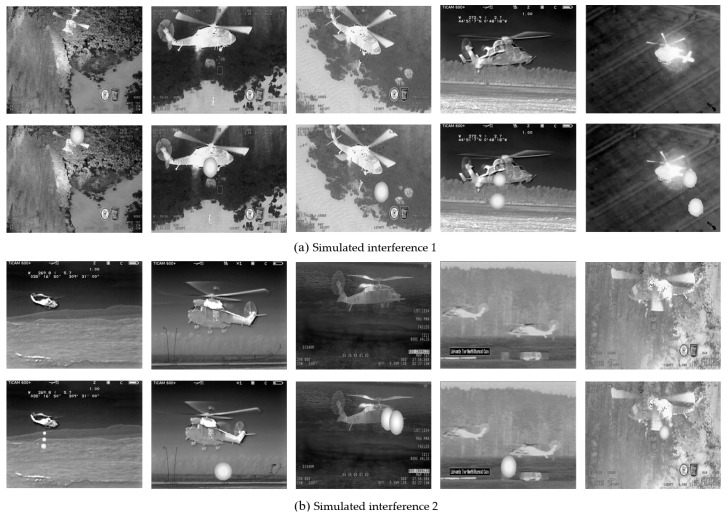
Infrared aircraft image with simulated interference under ground background.

The experimental data in this paper are sourced from the helicopter subset of the publicly available infrared dataset LSOTB-TIR [46], consisting of a total of 22,043 images. The background can be categorized into sky and ground, while the helicopter’s flight postures include two types: lateral and backward. The movement directions include approaching and receding. Since this portion of the dataset does not include manually released countermeasures, this paper simulates the radiation, shape variations, and movement of countermeasures and superimposes them onto the helicopter targets to create an infrared helicopter dataset under countermeasure conditions, as shown in Figure 8 and Figure 9. Figure 8 represents the partial data of the sky background, and Figure 9 represents the partial data of the ground background. The first row shows the original infrared aircraft data from LSOTB-TIR, while the second row displays the infrared aircraft data with artificially added interference. The added interference follows these rules:

(1) The interference radiation is modeled using a Gaussian distribution, with the highest gray value at the center and decreasing gradually outward.
(2) After release, the interference increases in size firstly, then decreases, while gradually moving away from the aircraft.
(3) The shape is modeled using a circular equation.

Using Labelme 3.16.7 software, annotations are created by generating a JSON file that includes target pixel category information and location details. For infrared aircraft targets under interference occlusion, the annotations are categorized into four classes with different colors: red for the head, green for the tail, yellow for the propeller, and blue for interference. The dataset is divided into training, validation, and test sets in a ratio of 8:1:1.

### 3.3. Data Analysis

#### 3.3.1. Evaluation Metrics

This paper uses mean intersection over union (MIoU), mean pixel accuracy (MPA), and mean recall (MR) as evaluation metrics for semantic segmentation algorithms.

(1) MIoU

MIoU is calculated by first computing the intersection over union (IoU) for each category, and then taking the average of the IoUs across all categories. The calculation formula is shown in Equation (15):

(15) $$\mathrm{MIoU}=\frac{1}{n+1}\sum_{i=0}^{n}\frac{p_{ii}}{\sum_{j=0}^{n}p_{ij}+\sum_{j=0}^{n}p_{ji}-p_{ii}}$$

(2) MPA

MPA is calculated by first computing the pixel accuracy for each category and then taking the average of the pixel accuracies across all categories. The calculation formula is shown in Equation (16):

(16) $$\mathrm{MPA}=\frac{1}{n+1}\sum_{i=0}^{n}\frac{p_{ii}}{\sum_{j=0}^{n}p_{ij}}$$

(3) MR

MR is calculated by first computing the recall for each category and then taking the average of the recalls across all categories. The calculation formula is shown in Equation (17):

(17) $$\mathrm{MR}=\frac{1}{n+1}\sum_{i=0}^{n}\frac{p_{ii}}{\sum_{j=0}^{n}p_{ji}+p_{ii}}$$

From Equations (15) to (17), i,j represent the category in the dataset, n is the number of categories in the dataset, n+1 denotes the total number of categories including labels and background, $p_{ii}$ represents the number of pixels that are actually of class i and are also predicted to be of class i, $p_{ij}$ is the number of pixels that are actually of class i but are predicted to be of class j, and $p_{ji}$ denotes the number of pixels that are actually of class j but are predicted to be of class i.

#### 3.3.2. Ablation Study

(1) Overall Quantitative Analysis

To validate the effectiveness of the designed location attention feature fusion network, hybrid atrous spatial pyramid pooling module, and dice loss in the semantic segmentation algorithm, this paper conducts ablation experiments using the HRNetv2 semantic segmentation algorithm as the basic framework. The experimental results are shown in Table 1.

1) To validate the effectiveness of the LAFFN module, this paper conducts comparative experiments by adding the LAFFN module to the HRNetv2 base algorithm. Compared to the HRNetv2 base algorithm, the MIoU improves by 0.22%, the MPA improves by 0.7%, and the MR improves by 0.21%, indicating that the LAFFN can enhance the segmentation performance of the algorithm.

2) To validate the effectiveness of the HASPP module, this paper conducts comparative experiments by adding the HASPP module to the HRNetv2 base algorithm. Compared to the HRNetv2 base algorithm, the MIoU improves by 0.25%, the MPA improves by 0.97%, and the MR improves by 0.30%, indicating that the HASPP module can enhance the segmentation performance of the algorithm.

3) To validate the effectiveness of the dice loss function, this paper conducts experiments by adding the dice loss function to the HRNetv2 base algorithm. Compared to using only the cross-entropy loss function in the HRNetv2 base algorithm, the MIoU improves by 2.35%, the MPA improves by 2.07%, and the MR improves by 1.25%, indicating that the dice loss function can enhance the segmentation performance of the algorithm.

4) To validate the effectiveness of the combined impact of the three measures, this paper conducts experiments by adding all three measures to the HRNetv2 base algorithm. Compared to the HRNetv2 base algorithm, the MIoU improves by 2.69%, the MPA improves by 2.68%, and the MR improves by 1.57% under the combined effect of the three measures. These results are higher than when each measure is used individually, indicating that the combined effect of the three measures can better enhance the segmentation performance of the algorithm.

(2) Effect of HASPP

To verify that the HASPP module has better detection performance compared to the ASPP module, this paper conducts comparative experiments by adding each of the two modules separately to the HRNetv2 algorithm. The experimental results are shown in Table 2. It can be observed that, compared to adding the ASPP module, adding the HASPP module to the HRNetv2 base algorithm results in an increase of 0.23% in MIoU, 0.82% in MPA, and 0.21% in MR. This indicates that the HASPP module designed in this paper can better enhance the detection performance of the algorithm compared to the ASPP module.

(3) Effect of LAM

**Figure 10:**
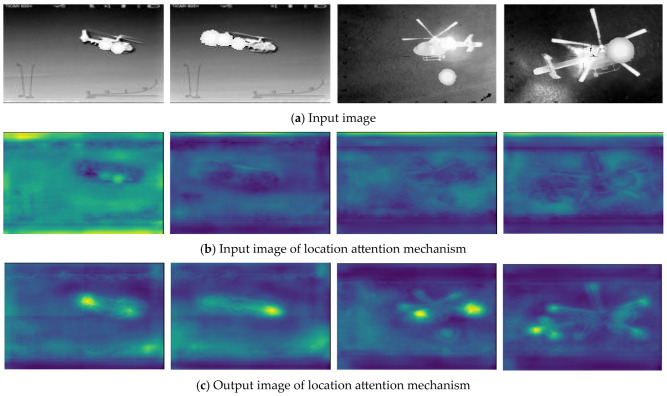
Visual effect of location attention mechanism.

To validate the enhancement effect of the location attention mechanism (LAM) on target feature information in this algorithm, a visual analysis of the input and output feature maps of the location attention mechanism is conducted, as shown in Figure 10. The first row represents the original images, the second row shows the feature maps input to the location attention mechanism, and the third row displays the output feature maps from the location attention mechanism. From the figure, it can be seen that after introducing the location attention mechanism:

1) In the first column, the head and tail features of the target are enhanced.

2) In the second column, the head features of the target are enhanced.

3) In the third column, the head, tail, and propeller features of the target are enhanced.

4) In the fourth column, the tail and propeller features of the target are enhanced.

Therefore, it can be concluded that introducing the location attention mechanism enhances the representation of aircraft features and highlights the information of specific target parts.

(4) Visual Segmentation Results for Three Measures

**Figure 11:**
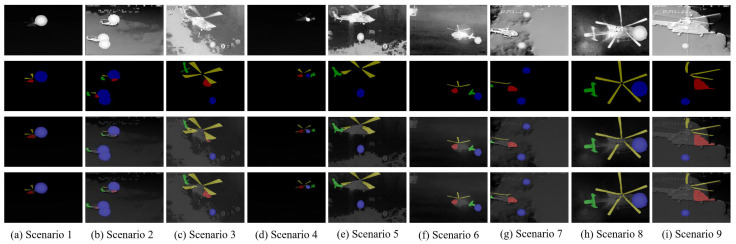
The comparison between HRNetv2 and HRNetv2+LAFFN for segmentation results.

Figure 11 shows the visualization results of the ablation experiments for HRNetv2 and HRNetv2+LAFFN. The first row displays the source images, the second row shows the ground truth labels, the third row presents the segmentation results of HRNetv2, and the fourth row shows the segmentation results of HRNetv2+LAFFN. It can be seen that the segmentation results of the HRNetv2+LAFFN algorithm are closer to the ground truth. For example, in Figure 11b, the aircraft head is more accurately segmented, and in Figure 11h, the propeller is more completely segmented using the HRNetv2+LAFFN algorithm.

**Figure 12:**
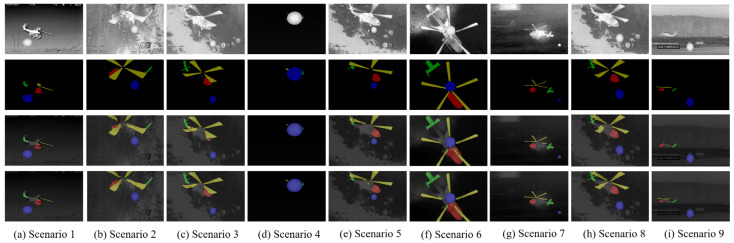
The comparison between HRNetv2 and HRNetv2+HASPP for segmentation results.

Figure 12 shows the visualization results of the ablation experiments for HRNetv2 and HRNetv2+HASPP. The first row displays the source images, the second row shows the ground truth labels, the third row presents the segmentation results of HRNetv2, and the fourth row shows the segmentation results of HRNetv2+HASPP. It can be seen that the segmentation results of the HRNetv2+HASPP algorithm are closer to the ground truth. For instance, in Figure 12c, the aircraft head, in Figure 12e, the aircraft tail, and in Figure 12h, the propeller, are more completely segmented using the HRNetv2+HASPP algorithm.

**Figure 13:**
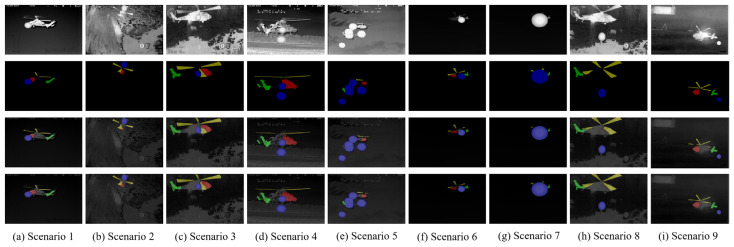
The comparison between HRNetv2 and HRNetv2+dice loss for segmentation results.

Figure 13 shows the visualized images from the ablation experiment comparing HRNetv2 and HRNetv2 with dice loss. The first row presents the source images, the second row shows the ground truth labels, the third row illustrates the segmentation results of HRNetv2, and the fourth row displays the segmentation results of HRNetv2 with dice loss. It is evident that the segmentation results of HRNetv2 with dice loss are closer to the ground truth. For instance, in Figure 13b, the aircraft head, in Figure 13c, the propeller, and in Figure 13e, the aircraft tail are more completely segmented by the HRNetv2 with dice loss.

#### 3.3.3. Comparative Experiments of Different Algorithms

Comparative experiments are conducted between HFSSNet designed in this paper and the semantic segmentation algorithms FCN [4], Improved U-Net [23], PSPNet [25], Improved DeepLabv3+ [12], Segformer [47], HRNetv2 [31], and DDRNet [48]. The results are shown in Table 3. It can be seen that the HFSSNet algorithm achieves the MIoU of 92.74%, MPA of 96.34%, and MR of 96.19% in the infrared aircraft segmentation task, all of which are higher than those of the other semantic segmentation algorithms.

**Figure 14:**
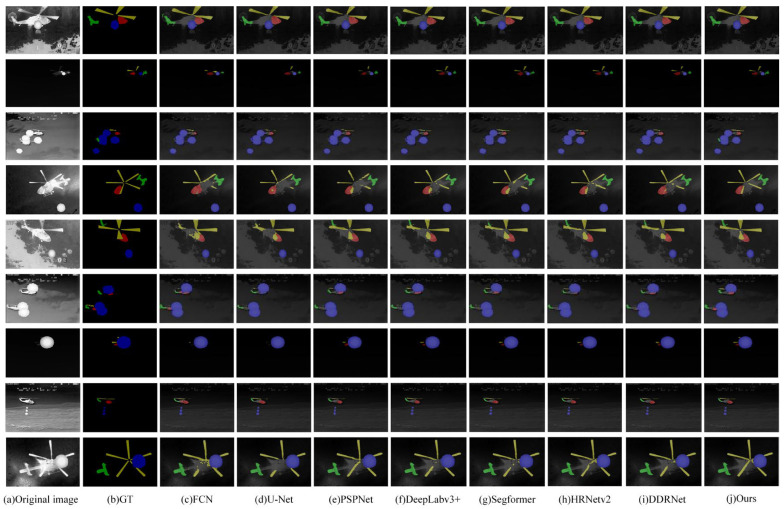
The segmentation results of different algorithms.

The visualization results of different semantic segmentation algorithms are shown in Figure 14. In the first row of data, all algorithms detected the aircraft head, tail, propeller, and interference. However, the results from the algorithm proposed in this paper are closer to the ground truth. Observing the detection results for the aircraft tail in the second and third rows of data, it can be seen from the second row that all algorithms can detect the infrared aircraft tail; however, the segmentation result of the proposed algorithm is closer to the ground truth. From the third row of data, it is evident that the proposed algorithm is able to detect the infrared aircraft tail, while the other algorithms fail to detect it. Observing the detection results for the aircraft head and propeller in the fourth and fifth rows of data, it is clear that the proposed algorithm can effectively segment the head and propeller, while the segmentation results of the other algorithms show a significant discrepancy compared to the ground truth. In the sixth row of data, the proposed algorithm correctly detects the aircraft head, tail, and propeller, whereas the other algorithms exhibit missed detections in their results. In the seventh row of data, compared to other algorithms, the proposed algorithm not only correctly detects the aircraft head and propeller, but also maintains a relatively complete structure. Observing the detection results for the aircraft propeller in the eighth and ninth rows of data, it can be seen from the eighth row that all algorithms can detect the propeller, but the proposed algorithm produces a more complete segmentation of the right-side propeller. From the ninth row, it is evident that the proposed algorithm finely segments the small left-side segments of the propeller affected by interference, while the segmentation results of the other algorithms are incomplete.

## 4. Conclusions

In order to achieve infrared aircraft detection under interference conditions, this paper proposes an infrared aircraft detection algorithm based on high-resolution feature-enhanced semantic segmentation network. The algorithm designs a location attention feature fusion network, which uses a location attention mechanism to obtain correlation weights between pixels at different locations, thereby enhancing the feature map of the current level. This enhanced feature map is then fused with a high-level feature map rich in semantic information to achieve feature enhancement of the target across different resolution feature layers. At the same time, a hybrid atrous spatial pyramid pooling module is designed to further enhance target features by capturing contextual information of the target within the same resolution feature layer, without losing feature information. In addition, by introducing the dice loss function, the algorithm deeply explores foreground information to fully learn from the existing samples. This paper constructs an infrared aircraft detection algorithm based on high-resolution feature-enhanced semantic segmentation network, which combines the location attention feature fusion network, the hybrid atrous spatial pyramid pooling module, the dice loss function, and a network that maintains the resolution of feature maps. Ablation experiments demonstrate the effectiveness of the three proposed improvements, while comparative experiments with different algorithms show the performance superiority of the proposed algorithm in infrared aircraft target detection under interference conditions. In future research, lightweight design of structures like LAFFN and HASPP could be considered to reduce the algorithm’s demand for computational resources and storage space, thereby enhancing the practical engineering value of the detection algorithm.

## 5. Patents

The research results of this article have been applied for a national invention patent in China (No. 202410820709.X).

## Figures and Tables

**Table 1:** Ablation analysis.

HRNetv2.	LAFFN	HASPP	Dice loss	MIoU/%	MPA/%	MR/%
√				90.05	93.66	94.62
√	√			90.27	94.36	94.83
√		√		90.30	94.63	94.92
√			√	92.40	95.73	95.87
√	√	√	√	92.74	96.34	96.19

**Table 2:** Verification results of ASPP and HASPP modules.

HRNetv2	ASPP	HASPP	MIoU/%	MPA/%	MR/%
√			90.05	93.66	94.62
√	√		90.07	93.81	94.71
√		√	90.30	94.63	94.92

**Table 3:** Segmentation results of different models.

Model	MIoU/%	MPA/%	MR/%
FCN	77.00	86.44	88.76
Improved U-Net	82.29	88.01	91.67
PSPNet	85.63	90.63	93.18
Improved DeepLabv3+	88.20	92.78	93.92
Segformer	89.49	93.10	94.51
HRNetv2	90.05	93.66	94.62
DDRNet	87.09	91.81	93.83
Ours	92.74	96.34	96.19

## Data Availability

Data in this article can be downloaded at https://github.com/QiaoLiuHit/LSOTB-TIR (accessed on 25 November 2020).
